# Clinical Usefulness of Susceptibility Breakpoints for Yeasts in the Treatment of Candidemia: A Noninterventional Study

**DOI:** 10.3390/jof6020076

**Published:** 2020-06-02

**Authors:** Cornelia Lass-Flörl, Robert Krause, Birgit Willinger, Peter Starzengruber, Petra Decristoforo, Sabrina Neururer, Peter Kreidl, Maria Aigner

**Affiliations:** 1 Institute of Hygiene and Medical Microbiology, Medical University of Innsbruck, 6020 Innsbruck, Austria; petra.decristoforo@tirol-kliniken.at (P.D.); peter.kreidl@i-med.ac.at (P.K.); maria.aigner@i-med.ac.at (M.A.); 2 Section of Infectious Diseases and Tropical Medicine, Medical University of Graz, 8036 Graz, Austria; robert.krause@medunigraz.at; 3 Division of Clinical Microbiology, Department of Laboratory Medicine, Medical University of Vienna, 1090 Vienna, Austria; birgit.willinger@meduniwien.ac.at (B.W.); peter.starzengruber@meduniwien.ac.at (P.S.); 4 Department of Medical Statistics, Informatics and Health Economy, Medical University of Innsbruck, 6020 Innsbruck, Austria; Sabrina.Neururer@i-med.ac.at

**Keywords:** antifungal susceptibility testing, clinical breakpoints, MIC, *Candida* species, in vitro versus in vivo outcome

## Abstract

This prospective noninterventional study evaluated whether antifungal susceptibility data (MIC) provided for *Candida* clinical isolates on the basis of recently established breakpoints are taken into account by clinicians to guide their treatment decision making process, and assessed the response in MIC- and non-MIC-based treatment groups. During a six month period, the usage of systemic antifungals was recorded in detail and compared with mycological data (*Candida* species and MICs) in candidemia patients. Patients were assigned to a susceptible or resistant infection group based on European Committee on Antimicrobial Susceptibility Testing (EUCAST) breakpoints; treatment decisions were under the professional discretion of the treating physicians. 123 patients were evaluated with *Candida albicans* accounting for 59%, *Candida glabrata* for 19%, *Candida parapsilosis* for 15%, *Candida tropicalis* for 4% and *Candida krusei* for 3%. Antifungal treatment correlated with species and MICs in 80% (*n* = 99 patients), high MICs and species-dependent guideline recommendations were ignored in 20% (*n* = 24 patients); the overall outcome of candidemia cases in our study population was excellent, as by day 14, all patients were cleared from fungal blood stream infection (mean 5.6 days, range 2–12). The current variability in antifungal usage and the delay in initiating appropriate therapy indicate a need for antifungal stewardship to improve the management of invasive fungal infections.

## 1. Introduction

*Candida* are the fourth most commonly encountered nosocomial pathogen in bloodstream infections (7–10% of isolates) [1,2]. Invasive candidiasis is associated with attributable mortality rates between 30 and 60%. Although *Candida albicans* is the predominant species, in recent years, there was a shift toward the isolation of other species, such as *C. glabrata, C. tropicalis, C. parapsilosis*, and *C. krusei* [3,4].

Overall, clinical resistance of *Candida* to established antifungal agents is rare and mostly seen for non-*albicans* species in critically ill and/or immunocompromised patients [3,4,5]. However, mounting evidence suggests that acquired resistance may be an emerging and underdiagnosed entity. Consequently, in vitro susceptibility testing of *Candida* isolates has become part of the clinical routine in some clinics to allow for tailored antifungal therapies [6,7].

Both the European Committee of Antimicrobial Susceptibility Testing (EUCAST) and the United States Clinical and Laboratory Standards Institute (CLSI) have developed and standardized broth microdilution methods for the in vitro susceptibility testing of yeasts [8,9]. Clinical cut off values for a variety of *Candida* species and antifungal agents are published by EUCAST [10]. Pfaller et al. underline the importance of molecular, clinical and microbiological data to meet species-specific criteria [11]. However, as these breakpoints have not been established for the clinical routine until recently, it is presently unclear whether antifungal susceptibility data (MICs) provided on microbiological laboratory reports prompt clinicians to reconsider and change the administered antifungal regimen. Thus, the clinical value of this laboratory information is under debate. Furthermore, the data available indicate that there may be a relationship between in vitro resistance and clinical failure, but not between in vitro susceptible results and therapeutic success [12].

The primary objective of this noninterventional study (NIS) was to evaluate whether antifungal susceptibility data provided for *Candida* clinical isolates on the basis of recently established breakpoints are taken into account by clinicians to guide their therapeutic decision making process. The secondary objective was to evaluate the response in MIC- and non-MIC-based treatment groups, by assessing the clinical outcome of patients in whom the first-line antifungal drug regimen was maintained despite classifying the causative *Candida* strain as resistant, and of those in whom the therapy was switched as a direct response to the obtained MIC data provided.

## 2. Materials and Methods

### 2.1. Patient Enrollment and Antifungal Susceptibility Data

This prospective multicenter NIS evaluated the clinical value of MICs and clinical breakpoints in the management of candidemia patients undergoing treatment with systemic antifungals (ethical votum AN2016-0118 363/4.7). The study population consisted of male/female patients at the age > 18 years suffering from documented candidemia requiring systemic antifungal therapy. Proven mycological EORTC/MSG (European Organisation for Research and Treatment of Cancer/Mycoses Study Group) criteria were specified as the diagnostic gold standard [13]. Hence, invasive *Candida* infection was defined as a blood culture that yields yeast cells via microscopy and culture. All fungal isolates obtained were identified to species level by standard laboratory methods and in vitro susceptibility testing was done according to EUCAST. Patients were assigned to a susceptible or resistant infection group based on EUCAST breakpoints [10].

During a six month period, eligible patients were recruited in the order of admittance to the study centers (Institute of Hygiene and Medical Microbiology, Medical University of Innsbruck, Section of Infectious Diseases and Tropical Medicine, Medical University of Graz, and Division of Clinical Microbiology, Department of Laboratory Medicine, Medical University of Vienna), and individuals derived from all hospital units. The decision of antifungal drug and when to use it was made under the professional discretion of the treating physician and followed local guidelines. In each case of positive blood cultures, the treating physician was requested to complete clinical data using a case report form (CRF) and the following case-based information was obtained: Age, gender, medical history, concomitant diseases, outcomes and antifungal treatment medications; the type and location of the *Candida* infection, the diagnostic methods used, species and MICs. Fungal blood clearance at day 14 defined the resolution of an infection; fungal presence at the site from infection despite antifungal treatment was defined as failure. The patients’ medical data for scientific purposes were processed and analyzed in a pseudonymized form. The species and MIC of the causative *Candida* isolates and any antifungal treatment given were recorded in detail. At the end of systemic antifungal treatment, the overall response was assessed according to the medical judgment of the physician in charge using the definitions mentioned above. The mycology lab did not explicitly point to prominent species and MICs. Physicians were free to comment the reasons of drug switches; species, MIC, and non-MIC-based treatment modalities were compared independently and matched with antifungals applied.

### 2.2. Statistical Analyses

A formal sample size calculation was not performed, but a minimum sample size of 100 patients was targeted and the study cohort was grouped according whether or not a switch of antifungals was made on basis of species and susceptibility results for the *Candida* isolate. We evaluated patients suffering from susceptible and resistant isolates and subsequent treatment. Patients in whom the antifungal therapy was changed because of intolerance, side effects or other reasons were excluded. While all quantitative values were expressed as median (range), absolute and relative frequencies were given for qualitative values. The Kolmogorov–Smirnov test was used for testing for normal distribution. The Mann–Whitney U test and Pearson’s Chi Square test were performed to analyze differences in demographic characteristics and outcome variables. *p* values < 0.05 were considered statistically significant. All statistical analysis was conducted with SPSS Version 25. (IBM Corp. Released 2017. IBM SPSS Statistics for Windows, Version 25.0. Armonk, NY, USA: IBM Corp.)

Quality controls of data were performed to ensure the accuracy and reliability of data. For this purpose, a person not otherwise involved in the NIS spot checked the CRFs and verified the entries against the source data. All patient data were recorded in paper CRFs, which were specifically designed to meet the data recording requirements of the present NIS.

## 3. Results

Overall, data from 144 clinical cases were collected; 21 cases were deleted as records were not complete. Hence, full data of 123 patients were evaluated. *C. albicans* accounted for 59% (*n* = 72), *C. glabrata* for 19% (*n* = 24), *C. parapsilosis* for 15% (*n* = 18), *C. tropicalis* for 4% (*n* = 5) and *C. krusei* for 3% (*n* = 4). Species distribution, MICs and susceptible and resistance classification are given in Table 1.

The study group consisted of 70 male and 53 female patients (mean age 63 years, range 22–91); 61 patients were from ICUs, all patients were non-neutropenic, see Table 2. In all study centers, fungal diagnosis followed the same algorithm, which include the immediate information of any positive fungal growth of yeasts at day 1, followed by species identification and MIC data between days 2 to 3.

**Figure 1:**
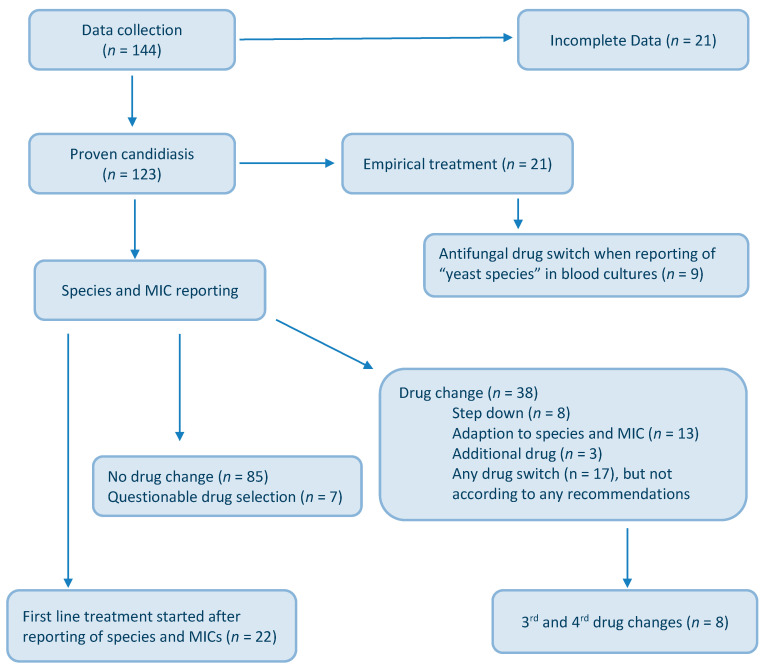
Flow chart of patients with proven candidiasis and antifungal treatment.

Figure 1 gives an overview on patients enrolled, antifungal drugs applied and treatment switches. An amount of 69% (*n* = 85) of patients received one antifungal drug throughout the treatment course, while 31% (*n* = 38) underwent a switch. The reasons of drug replacement were multiple and included, among others, step down therapy, adaption to species and MIC, and reporting of “yeast being present in the human specimen”.

A total of 43% (*n* = 53) of patients were under short-term empirical treatment at time-point of yeasts being detected, as shown in Table 2. Based on the lab information, yeasts growing in the blood culture led to an immediate switch of the existing antifungal drug in 7% (*n* = 9), and another drug was added in 2% (*n* = 3). The subsequent shift included the application of echinocandins, liposomal amphotericin B (l-AmB), and isavuconazole (ISA), despite not knowing the species and MICs at that moment. In 18% (*n* = 22) antifungal treatment started only when species and MIC were available, hence treatment started rather late. Overall, antifungal treatment was conform to species and MICs in 80% (*n* = 99 cases) of cases; high MICs and species-dependent guideline recommendations were ignored in 20% (*n* = 24 cases) of cases, as shown in Table 3; and another third or fourth switch was made in eight patients for various clinical reasons, including two cases of *C. albicans* candidemia and low MICs. Overall, the outcome of candidemia cases in our study population was excellent, as by day 14, all patients (*n* = 123) were clear of fungal blood stream infections (mean 5.6 days, range 2–12), independent of antifungal treatment applied and species involved. The eight cases with multiple drug switches suffered from *C. parapsilosis* (*n* = 4), *C. albicans* (*n* = 2), *C. krusei* (*n* = 2).

The statistical analyses showed that there was no difference in outcome based on empirical or targeted therapy (*p* = 0.881), MIC based treatment (*p* = 0.713), resistant or susceptible fungal categorization (*p* = 0.744), species involved (*p* = 0.570), switch to any antifungal (*p* = 0.187), sex (*p* = 0.259), age (*p* = 0.880), or ICU admittance (*p* = 0.744), as shown in Table 4.

## 4. Discussion

The present NIS is the first report documenting that antifungal treatment is in agreement with underlying species and MICs in 80% of cases. This multicenter study showed a high frequency of antifungal drug switches during the course of candidemia treatment. MIC- and non-MIC-based antifungal treatment regimens showed no significant difference in outcome, as all patients resulted in fungal free blood cultures after 14 days of antifungal therapy.

The frequency of *Candida species* causing candidemia depends on predisposing patients’ conditions, on antifungal agents prescribed, and local hospital-related factors being present [14]. In line with other reports, the most important species in our study were *C. albicans, C. glabrata,* and *C. parapsilosis*. 95% of *C. parapsilosis* and 100% of *C. glabrata* were documented to be resistant against anidulafungin and fluconazole, respectively.

The majority of patients (66%) received an echinocandin as first-line treatment, following the current guideline of the European Society for Clinical Microbiology and Infectious Diseases (ESCMID) [15]. An amount of 28% started with fluconazole, which clearly contrasts with the ESCMID guideline [15] but supports IDSA recommendations [16]. The echinocandins have emerged as preferred agents for candidemia and invasive candidiasis, with some exceptions. This recommendation is based on a good safety profile, a trend toward better outcome data, and the emergence of azole-resistant *Candida* species. Neither ESCMID [15] nor IDSA guidelines [16] differentiate between the three available echinocandins in their treatment recommendations.

Three major findings from this NIS were novel and of great surprise for us; first, the notification of a yeast being present in the blood culture prompted clinicians to change the present empirical antifungal treatment (mean 3.5 days, range 2–9); in the majority of cases, fluconazole was replaced by an echinocandin, or an additional antifungal was added, despite not knowing the species at that time-point. We speculate that clinicians interpreted the growth of yeasts as a failure of the underlying empirical treatment. Second, 22 patients received an antifungal treatment only after species identification and MICs were reported by the microbiology lab; which in turn means that antifungal treatment starts late in the course of candidemia. It is well known that the delayed administration of any antifungal treatment among patients with *Candida* bloodstream infections is associated with greater hospital mortality [17]. Third, species and MIC- based treatment regimens may only have a marginal influence on the outcome of candidemia; in our study, patients were successfully treated with fluconazole (high dosing, 10 mg/kg/body weight) even with the presence of fluconazole-resistant *C. glabrata*, which is in line with Eschenauer et al. who recommend a high dose of fluconazole for the treatment of *C. glabrata* infections, irrespective of MICs [18]. Ghanem-Zoubi et al. underline the need for high dosing in patients infected with high MIC-*Candida* species [19]. *C. parapsilosis* cleared well from blood stream infections under echinocandin therapy and finally, despite anidulafungin resistance being present, treatment with anidulafungin or with another echinocandin was successful after 14 days of therapy. Our data are supported by Kontoyiannis et al. [20], who showed that anidulafungin is effective for the treatment of *C. parapsilosis* candidemia; in addition, Wu et al. [21], demonstrated a lower 30-day mortality rate for *C. parapsilosis* when compared to *C. albicans* candidemia. During the study period, eight patients underwent another third or fourth antifungal drug switch, however these patients were finally clear of the yeasts within 14 days of observation. The latter group included two cases with *C. albicans* candidemia. Antifungal step down management or de-escalation treatment based on species was rarely implemented in our study population with the reason of them not being well understood. The reporting of susceptible strains led to no antifungal changes in the majority of patients.

Up to now, no single trial has demonstrated clear superiority of one therapeutic agent over another in candidemia, and careful analysis of clinical data sometimes leads to conflicting conclusions [22,23,24]. These facts may also explain the inconsistent approach of species and MIC-guided treatment in invasive fungal infections.

The notification of species and MICs of any underlying fungal pathogen should support treatment adjustment according to in vitro data. Hence, ESCMID as well as IDSA guideline recommend not to treat *C. parapsilosis* infections with an echinocandin; *C. parapsilosis* demonstrates innately higher MICs to the echinocandins than most other *Candida* species, which raises the concern that *C. parapsilosis* may be less responsive to the echinocandins [15,16]. In our dataset, 95% of *C. parapsilosis* strains were designated to be in vitro resistant. The same is valid for fluconazole and *C. glabrata;* however, the experience of this NIS was that the influence of MICs on overall outcome may be low. A total of 20% of patients (*n* = 24 patients) enrolled were not treated according to these recommendations, but controversially the outcome was quite good. These outcome data are potentially grounded by the fact that the majority of patients were not severely ill, and as we included patients from all wards, only 49% were from the ICU. Table 3 gives detailed information on responsible fungal pathogens involved and treatment regimens applied. Several unresolved questions exist; however, the patients’ well-being led the treating physicians not to change the underlying drug management. It remains unclear whether adherence to current ESCMID guidelines would have impacted on the patients’ outcome. So far, it seems that susceptibility testing data have value, but far less than what was thought. Similar data were observed by Ghrenassia et al. [25], who assessed the outcome of candidemia in clinically ill patients. Our findings are supported by the 90-60 rule which underlines that infections due to susceptible and resistant isolates respond to appropriate therapy in ~90% and ~60%, respectively [26]. Various other factors may influence the outcome (e.g., drug pharmacokinetics, drug delivery to the site of infection, treatment of the site of infection, host response) [27,28], and it is obvious how one or more of these aspects might outweigh the impact of MIC results. Susceptibility data could point out that a single MIC measurement is not sufficiently precise to warrant the outcome, or that clinical breakpoints do not sufficiently reflect the complex in vivo situations. Contrasting data are shown by Ko, [29] et al., displaying increased fluconazole MICs being associated with a poor outcome. However, the authors do not comment on fluconazole dosing (high or standard) regimens applied.

Due to the limited cases, we cannot comment on whether the various echinocandins display a different outcome in terms of MICs and *C. parapsilosis;* these three lipopeptides were multiply applied as second-line treatment, irrespective of anidulafungin resistance being present or not (Table 3).

Testing for azole susceptibility is recommended for all bloodstream and other clinically relevant *Candida* isolates, and testing for echinocandin susceptibility should be considered in patients who had prior treatment with an echinocandin and among those who have infection with *C. glabrata* or *C. parapsilosis*. Reflecting the clinical problem, IDSA declared this advice with a strong recommendation but a low-quality of evidence, [16] and our study data may support this main message. The superior outcome of C. glabrata and fluconazole treatment in our study may be related to the fact that the highest dosing of fluconazole was applied in all patients.

Limitations of this study are the small number of patients infected with drug-resistant yeasts, an underlying heterogeneous patient population with lack of severely ill patients, and not taking into account catheter exchanges. Non-neutropenic patients who have candidemia may clear from fungal bloodstream infections immediately after catheter removal [30,31]; in addition, early Central venous catheter withdrawal was associated with 30-day mortality [32]. Our key message is that all of our patients investigated fall into this classification; hence in the future, a greater sample size is required to determine more reliably the association of species distribution and MICs in the outcome of antifungal treatment.

In conclusion, antifungal treatment was 80% correlated with species and MICs. The variability of antifungal drug use and the delay in initiating appropriate therapy (20%) indicate a need for antifungal stewardship in order to improve the management of invasive fungal infections. Important questions of how to best implement MIC data into the clinical routine remain unanswered and require further clinical studies.

## Figures and Tables

**Table 1:** *Candida* species and susceptibility classification (%) according to EUCAST guideline [10].

Species	Number	Anidulafungin		Fluconazole		Voriconazole		Posaconazole		Amphotericin B	
		S	R	S	R	S	R	S	R	S	R
*C. albicans*	72	96	4	100	0	100	0	95	5	100	0
*C. glabrata*	24	100	0	0	100	80	20	45	55	100	0
*C. parapsilosis*	18	5	95	78	22	100	0	94	6	100	0
*C. tropicalis*	5	100	0	100	0	100	0	100	0	100	0
*C. krusei*	4	100	0	0	100	100	0	50	50	100	0

S, susceptible strains expressed in %; R, resistant strains expressed in %.

**Table 2:** Demographic and clinical characteristics of 123 patients with proven yeasts infections receiving antifungal therapy.

Variables	Number of Patients (%)
Demographic parameters	
Age, range	Median 63 years, range 22–91
Male gender	70 (75%)
Medical ward	39 (32%)
Surgical ward	62 (50%)
Others	22 (18%)
ICU stay (overall)	61 (49%)
Treatment	
Empirical treatment	53 (43%)
Antifungal drugs used as first-line treatment	
Caspofungin	38 (31%)
Fluconazole	35 (28%)
Anidulafungin	31 (25%)
Micafungin	12 (10%)
Voriconazole	5 (4%)
Posaconazole	1 (1%)
Fluconazole and Flucytosine	1 (1%)
Days until fungal clearance	Median 5.6 days, range 2–12
Outcome	
14-day fungal free blood cultures	123 (100%)

**Table 3:** Antifungal treatment modalities and species and MIC values provided at the study centers.

Species (Patients)	Antifungal Drug Resistance	Primary Therapy (Patients)	Antifungal Switch	14-Day Fungal Free Blood Cultures	Open Questions Based on International Guideline Recommendations and *Candida* Species and MIC Values Reported
*C. albicans* (*n* = 3)	ANI	CAS	MICA & l-AmB ISA	✓	The switch from CAS to MICA is unclear?
		CAS		✓	The application of ISA, which is not licensed for Candida therapy is unclear?
		CAS		✓	The application of CAS despite ANI resistance is unclear?
*C. glabrata* (*n* = 14)	FLU ANI	FLU (7)	FLU & ANI CAS	✓	The application of FLU in C. glabrata and FLU resistance being present is unclear?
		FLU		✓	Why not stopping FLU when adding ANI?
		ANI		✓	Why a change from ANI to CAS in such case?
		ANI (5)		✓	The application of ANI despite ANI resistance being present is unclear?
*C. parapsilosis* (*n* = 6)	ANI ANI/FLU FLU	CAS	MICA	✓	The switch from CAS to MICA despite ANI resistance being present is unclear?
		ANI (2)		✓	ANI treatment despite ANI resistance being present?
		CAS		✓	CAS therapy despite ANI resistance?
		CAS		✓	Why CAS therapy despite ANI resistance?
		FLU		✓	The application of FLU despite FLU resistance being present is unclear?
*C. krusei* (*n* = 1)	FLU	MICA	CAS	✓	Why change from MIC to CAS in case of FLU resistance being present?

ANI, anidulafungin; CAS, caspofungin, MICA, micafungin; ISA, isavuconazole; FLU, fluconazole; l-AmB, liposomal amphotericin B.

**Table 4:** Univariate analyses of 14-day fungal free blood cultures elated to the various treatment variables.

Variables	Univariate *p* Value
Age	0.880
Gender	0.259
Empirical treatment	0.881
Targeted treatment	0.881
MIC (antifungal susceptibility data)-correlated treatment	0.713
MIC-non-correlated treatment	0.867
Antifungal drug switch	0.187
Intensive care unit	0.744
*Candida* species involved	0.570
Resistant isolates involved	0.721
Susceptible isolates involved	0.657
